# Effect of music on driving performance and physiological and psychological indicators: A systematic review and meta-analysis study

**DOI:** 10.34172/hpp.2023.32

**Published:** 2023-12-16

**Authors:** Morteza Ghojazadeh, Mehdi Farhoudi, Mahdi Rezaei, Sama Rahnemayan, Mahshad Narimani, Homayoun Sadeghi-Bazargani

**Affiliations:** ^1^Neurosciences Research Center (NSRC), Tabriz University of Medical Sciences, Tabriz, Iran; ^2^Road Traffic Injury Research Center, Tabriz University of Medical Sciences, Tabriz, Iran; ^3^Department of Biopsychology, Institute of Cognitive Neuroscience, Faculty of Psychology, Ruhr-University Bochum, Germany; ^4^Student Research Committee, Tabriz University of Medical Sciences, Tabriz, Iran

**Keywords:** Driving, Meta-analysis, Music, Physiologic, Psychologic, Digital epidemiology

## Abstract

**Background:**

Many studies have assessed the effect of music on driving. However, their results are very scattered and contradictory. Therefore, this systematic review is conducted to determine the effect of music on driving performance and drivers’ physiological and psychological indicators.

**Methods:**

Scopus, PubMed, and Web of Science databases were searched until July 2023. A manual search in Google Scholar for gray literature was conducted. The Simulation Research Rubric (SRR) tool was used to assess the reporting quality of the studies. Stata software (StataCorp, version 16) was used to perform a meta-analysis.

**Results:**

A total of 2650 records were identified. The findings of 19 studies were analyzed. Most of them were carried out in high-income countries (HICs) using simulators. The most frequently used music style was classic rock. The meta-analysis results indicated that music with high and medium volume increases the average driving speed, and music with low volume decreases it. Although music in every mood reduces the average reaction time, it positively reduces response delay and increases coherence. Music with high volume decreases the heart rate, but music with medium and low volume increases it. Listening to music increases the level of arousal and mental load.

**Conclusion:**

It was concluded that, in some indicators, listening to music has adverse effects on driving. However, in many indicators, music has a positive impact on improving driving safety. It is better to choose appropriate music for different driving conditions and to train the drivers about it.

## Introduction

 One significant contributing factor to worldwide social and economic issues is traffic accidents. Globally, traffic accidents claim the lives of over 1.3 million people annually, with automobile crashes accounting for the majority of deaths among children and young adults between the ages of 5 and 29. Car crashes are thought to have caused non-fatal but crippling injuries to 20‒50 million people. These injuries come at a heavy financial cost to the victims, their families, and society at large—roughly 3% of a nation’s yearly gross domestic product (GDP). This emphasizes the importance of examining driving procedures in greater detail, particularly when considering potential strategies to lower the number of casualties.^1,2^

 Listening to music while driving is one of the most important causes of distraction.^3,4^ Although in-car listening may seem trivial, it is evident that for 72%–100% of drivers, it has become an essential part of the driving experience.^5^ Since the turn of the millennium, the automobile has been the most preferred setting for listening to music.^6,7^ In fact, 75% of the time drivers spend behind the wheel, they listen to music.^8^

 Driving quality is affected by the driver’s attention, performance, and response. The way that people react also depends on their mood, and since music can change the listener’s mood, it affects the quality of driving.^9^ The effect of listening to music while driving, however, may be controversial.^10,11^ On the one hand, listening to music while driving can boost a driver’s level of arousal. Additionally, listening to music can enhance not only the driver’s driving quality but also their physiological performance. In particular, listening to music while driving is effective in controlling stress, calming emotions, and preventing driver drowsiness.^6,12,13^

 On the other hand, listening to music while driving could raise the driver’s mental workload index and, thus, impair their driving performance. In fact, both driving and listening to music compete for the driver’s limited cognitive capacity.^14^ According to a study by the American National Highway Traffic Safety Administration, in-vehicle driver distraction, such as listening to music, is responsible for 25% of traffic accidents.^15^

 Many researchers have attempted to measure the effect of listening to music using various valid scientific methods and tools, especially driving simulators.^6,8,11,12,14^ As a result, much information has been obtained about the mechanism and intensity of the effect of music on the quality and performance of driving, as well as the drivers themselves. First, it should be noted that the findings of these studies were dispersed throughout several scientific sources and were not coherent.^2^ Second, there is a lot of variation in the design and objectives of these studies, and finally, in many cases, significant contradictions are seen among the results of the studies.^16,17^ In this systematic review, we aim to collect and analyze these studies to effectively utilize them for decision-making and the design of additional interventions. Therefore, the current study aims to systematically review and meta-analyze the effect of music on driving performance and drivers’ physiological and psychological indicators.

## Methods

 The Preferred Reporting Items for Systematic Reviews and Meta-Analyses (PRISMA) guide was used.^18^ The protocol for this systematic review is available on https://www.crd.york.ac.uk/prospero/display_record.php?RecordID = 445865.

###  Study question (PICOTS)

*Participants:* Healthy adults between the ages of 18 and 60 with a valid driver’s license

*Intervention:* The effect of music or song (from any genre, at any volume, in any playback format, and regardless of whether the song was chosen by the individuals or by the researchers)

*Comparison:* No intervention, no music group, or condition

*Outcomes:* physiological indicators (like heart rate and heart rate variability), psychological indicators (such as mood, level of arousal, and mental workload), driving performance (such as reaction time, delay, and mean speed)

*Time:* No time restrictions

*Study design:* experimental studies (trials and semi-experimental studies) and studies conducted with simulators

 Studies were excluded if they had no control groups (no music), inappropriate study designs (cohorts, surveys), assessing the interaction with an in-vehicle music player, proceedings, or cases with data extraction restrictions.

###  Search strategy

 A qualified librarian built the search strategy with the guidance of a subject-matter expert (Supplementary file 1). The relevant studies were searched using related keywords and MeSH terms in PubMed, Scopus, Web of Science databases up to July 2023. To have a comprehensive search, Google Scholar was also manually searched for gray literature. References and cited lists of the relevant articles were also assessed for potentially pertinent studies.

###  Study selection

 First, all articles were reviewed by title, and ineligible ones were excluded. In the next steps, the abstract and the full text of the studies were evaluated. All steps of study selection were done independently by two authors. Disagreements and uncertainties were resolved through discussion and the help of a third reviewer. Endnote 20.2.1 reference manager software was used in all steps of study selection. The PRISMA 2020 flowchart was used to report the results of the selection and screening process.

###  Reporting Quality Assessment

 The reporting quality of all studies was assessed in the full text by two reviewers independently through the Simulation Research Rubric (SRR) tool. This tool was developed by Fey et al in 2015.^19^ The tool contains 16 items that are scored from 0 to 4. The lowest possible score was 0, and the highest score was 64. Two questions that had no relevance to the included studies were eliminated with the research team’s approval and 14 questions, ranging from 0 to 56, were used to evaluate the studies. The reporting quality of the three articles that were conducted on road (not in simulators or laboratories) was not assessed. Each article received a final evaluation score that was agreed upon by the two reviewers. The third reviewer was consulted in cases of disagreement between the two evaluators. No study was excluded from the study due to the quality assessment score.

###  Data extraction

 The data extraction form was designed in the Office Excel 2019 software (Available from: https://office.microsoft.com/excel). Extracted information in the form includes: first author, year of publication, first author affiliation (country), participants’ number, participants’ age (Mean /Median), participants’ sex, driving experience (year), listen to music (%), driving distance (km), experimental conditions, familiarization with the driving situation (Yes or No), test time (daytime or night), simulator control, simulator mobility (fixed or portable), simulator open source (Yes or No), simulator screen (Inch), simulator screen view, music number, music/test duration (minute), music selection (driver-selected or researcher-selected), music familiarity (Yes or No), music tempo (slow < 80 bpm, medium 80–120 bpm, or fast > 120 bpm), music volume (low ≤ 60 dB, medium 61–79 dB, high ≥ 80 dB), music lyrics (Yes or No), music style, outcome, outcome and standard deviation or standard error in music and no music groups, outcome significance (*P* < 0.05) (Yes or No), and outcome direction (positive or negative).

 As a test for this form, the data from three articles was first extracted, and the defects in the initial form were resolved. The data were extracted by two authors and checked by the second reviewer.

## Data Analysis

 In order to quantitatively analyze a number of indicators that had sufficient data, the random effect model meta-analysis statistical method was used due to high heterogeneity and this assumes that the true effect size could vary from one study to the next. The results of other indicators which quantitative analysis (meta-analysis) was not possible for them, were analyzed and reported descriptively. Stata software (StataCorp. 2019. Stata Statistical Software: Release 16. College Station, TX: StataCorp LLC.) was used to perform meta-analysis. Forest plots were used to report the results. I^2^ index was used to measure the heterogeneity. In this study, I^2^ less than 50% is considered low heterogeneity, I^2^ between 50 and 74 is considered medium heterogeneity, and I^2^ above 75% is considered high heterogeneity.^20^ Based on the volume and tempo of the music, subgroup analyzes was performed. In the studies in which indicators based on the volume or tempo of the music were not reported separately, in order to address the results and perform more appropriate analyses, the values of that index were repeated according to the number of possible situations. Egger’s regression test and funnel plot were applied to measure the publication bias at a significance level of 0.1%.^21^ In cases where there was a possibility of publication bias, the Trim and Fill test was used with the linear estimator method.

 The mean level of arousal index was reported using two different tools, including Russell, Weiss, and Mendelsohn’s AGRID questionnaire^22^ and the Mayer & Gaschke Brief Mood Introspection Scale (BMIS) questionnaire.^23^ The score of AGRID questionnaire (from 1 to 9) was converted to a BMIS score (12 to 48) using the proportion technique.

 Although there is a distinct difference between “movement time”, “response time”, and “reaction time”, few studies have not taken this into account. In these studies, “response time” and “reaction time”, were considered equal, but movement time was analyzed separately.

## Results

###  Study selection

**Figure 1:**
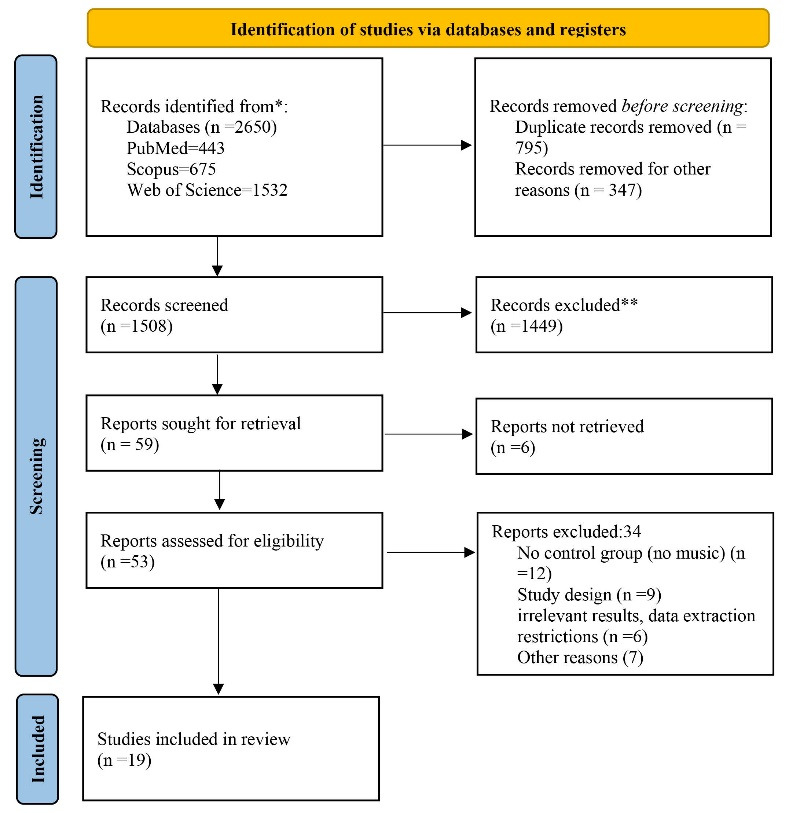
PRISMA 2020 flow diagram

 The search yielded 2650 papers, of which 795 were duplicates. After screening based on titles and abstracts, 1449 records were excluded. Six reports were not retrieved. Finally, 34 studies were excluded evaluating full texts based on exclusion criteria, and 19 studies (with 20 experiments) were included (Figure 1).^14,17,24-40^

###  Study characteristics

 The articles were published between 1999 and 2022 (median: 2013). Based on the World Bank classification, most of the studies were conducted in high-income countries (HICs) (80% of studies). A total of 987 individuals participated in the included studies. Participants’ average age was 23.9 years, with about 4.6 years of driving experience. Most of the studies used driving simulators (65%). In 62% of studies, the type of music was chosen by the researchers. All three tempo modes—fast, medium, and slow—were used in 10 of those studies that have mentioned music tempo conditions. In terms of music volume, high volume was the most repeated one (11 studies). Classic rock was the most selected type of music (in 60% of studies) (Table 1) (Supplementary file 2).

**Table 1:** Characteristics of conducted articles on different dimensions of the music effect on driving performance and its physiological and psychological indicators

**Dimension**	**Variable **	**Variable Level**	**Results**
Bibliometric information	Publication year (20)	Before 2010	6(30%)
		2010 to 2015	6(30%)
		2016 to 2020	5 (25%)
		2021 to 2023	3(15%)
	Countries (20)	Australia	2(10%)
		Brazil	1(5%)
		China	2(10%)
		France	3(15%)
		India	1(5%)
		Palestine	2(10%)
		Netherlands	2(10%)
		UK	3(15%)
		USA	4 (20%)
Participants characteristics	Participants Number (19)		987 (Male=444, /female 546)
	Participants Age (Mean) (19)		23.9 years
	Driving experience (Year)/Mean (12)		4.67 years
	listen to music (%)/Mean (6)		94.86 %
Procedure	Driving distance (KM) /Mean (3)		16.3 KM
	Experimental conditions (20)	Simulator	13 (65%)
		Road	3(15%)
		Laboratory	4 (20%)
	Familiarization with driving situation(18)	Yes	18 (100)
		NO	0
	Testing time (5)	Daylight	5 (100)
		Night	0
Simulator	Mobility (7)	Fixed	6(85.7)
		Portable	1(14.3)
	Open source (4)	Yes	4 (100)
	Screen size (Inch) (6)	Mean	25 Inch
	Screen view (5)	Mean	149.9
	Music number (15)	Median	5
	Music/test duration (minute) (13)	Mean	36 minutes
	Music Selection (21)	researcher-selected	13 (61.9%)
		driver-selected	5(23.8%)
		Both of them	3 (14.3%)
			
	Music Familiarity (16)	Yes	13 (81.2%)
		NO	3 (18.8%)
Music	Music Tempo (11)	Fast	10 (90.9%)
		Moderate	10 (90.9%)
		Slow	10 (90.9%)
	Music Volume (19)	High	11 (57.8%)
		Moderate	6(31.5%)
		Low	8(42.1%)
	Music Style (15)	Classic Rock	9 (60%)
		hard rock/heavy metal	4(26.6%)
		jazz/blues	4(26.6%)
		R&B/soul/funk	3(20%)
		Country	3(20%)
		TV	2 (13.3%)
		classical	2 (13.3%)
		Easy listening	2 (13.3%)
		Others	3(20%)
	Music lyrics (7)	Yes	5(71.4 %)
		NO	2(28.6%)

###  The effect of music on drivers’ performance

 In 17 experiments that showed the effect of music on various indicators of driving performance, data on 91 indicators were extracted. Music affected drivers’ performance; 62 indicators (68.1%) significantly, 26 indicators insignificantly (28.5%) and in three cases (3.4%) no significant level was reported. In terms of positive or negative impact, positive impact was reported in 49 indicators (53.8%), and negative impact was reported in 42 indicators (46.2%).

####  The effect of music on the average driving speed

**Figure 2:**
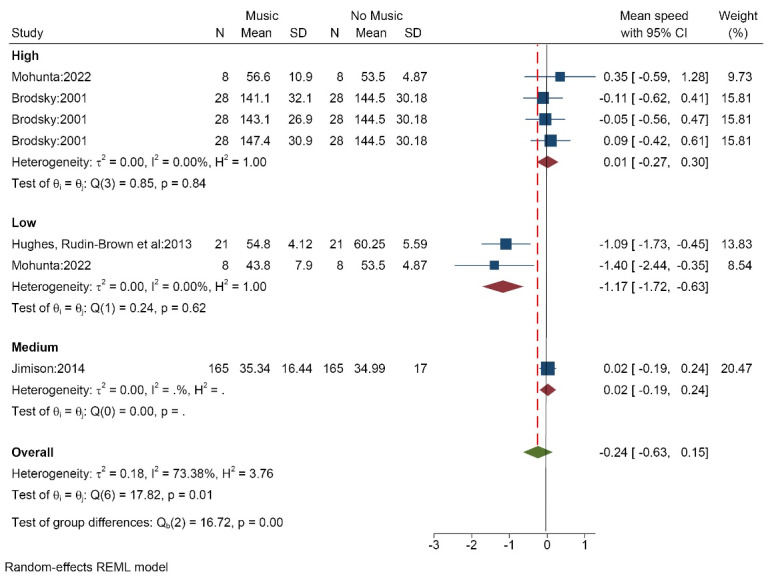
Effect of listening to music on average driving speed based on the music volume

 Four studies (7 data/conditions) with 222 participants (mean age of 31.57 years) measured the effect of music on average driving speed. Music was selected by the researchers. The results showed that high volume (SMD = 0.01 [-0.27, 0.30]) and medium volume (SMD = 0.02 [-0.19, 0.24]) music enhanced the average driving speed, while low volume music (SMD = -1.17 [-1.72, 0.63]) reduced it. Totally, music decreased the average driving speed (SMD = -0.24 [-0.63, 0.15]) (Figure 2). The results of the heterogeneity test showed that there is a medium level of heterogeneity (I^2^ = 73.3%). Per Egger’s test there is low probability of publication bias (*P* = 0.210, Z = -1.25) (Supplementary file 3, funnel plot A).

####  Effect of music on reaction/response time

**Figure 3:**
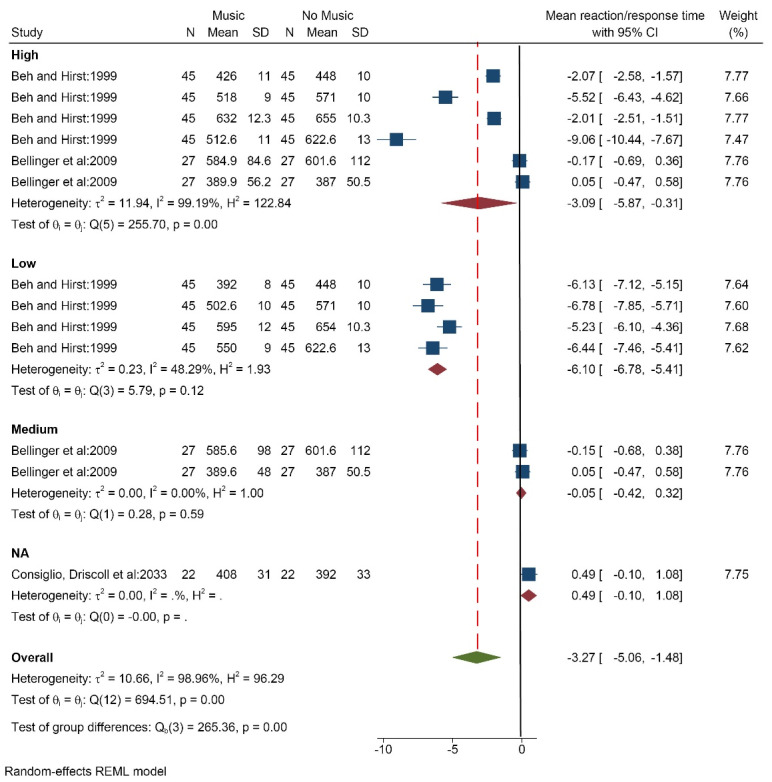
Effect of listening to music on the reaction/response time based on the music volume

 Three studies (13 data/conditions) with 92 participants (mean age of 20.6 years) measured the effect of music on mean reaction/response time. Music was selected by the researchers. The overall results showed that music in all three modes; high (SMD = -3.09 [-5.87, -0.31]), medium (SMD = -0.05 [-0.42, 0.32]), low (SMD = -6.10 [-6.78, -5.41]), and total (SMD = -3.27 [-5.06, -1.48]) caused decreased average response/reaction time (Figure 3). The results of the heterogeneity test showed that there is relatively high heterogeneity in the study results (I^2^ = 98.9%). There is a high probability of publication bias according to tests (Egger test *P* < 0.001, Z = -9.55) (Supplementary file 3, funnel plot B). The analysis based on music tempo showed similar results.

####  Effect of music on response delay

**Figure 4:**
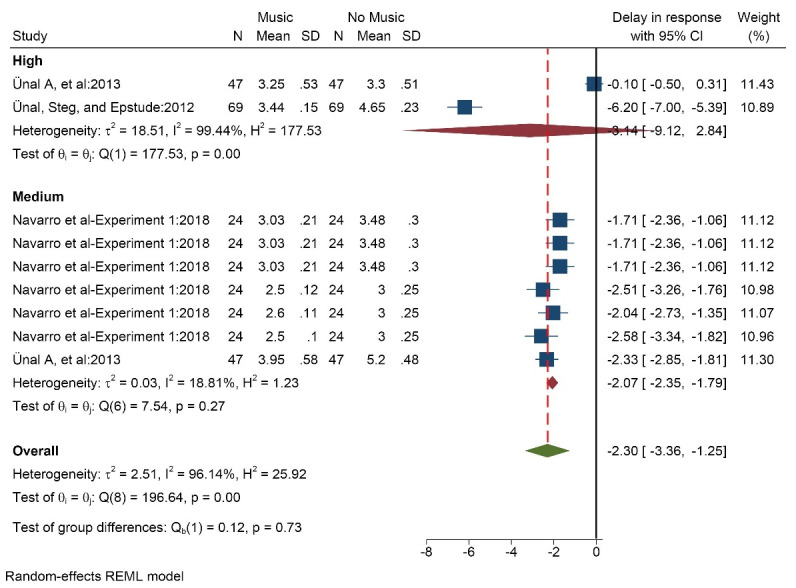
Effect of listening to music on the response delay based on the music volume

 Three studies (9 data/conditions) with 140 participants (mean age 22 years) measured the effect of music on response delay. Music was chosen by the participants. Music at both high volume (SMD = -3.14 [-9.12, 2.84]) and medium volume (SMD = -2.07 [-2.35, -1.79]) as well as in total (SMD = -2.30 [-3.36, -1.25]) reduced the response delay (Figure 4). The results of the heterogeneity test showed that there is a high degree of heterogeneity in the results of the studies (I^2^ = 96.1%). There is a high probability of publication bias (Egger test P < 0.005, Z = -2.78) based on tests (Supplementary file 3, funnel plot C). The results based on music tempo also indicated that, fast, medium, and slow modes reduced response delay (Supplementary file 3, funnel plot B).

####  Effect of music on coherence

**Figure 5:**
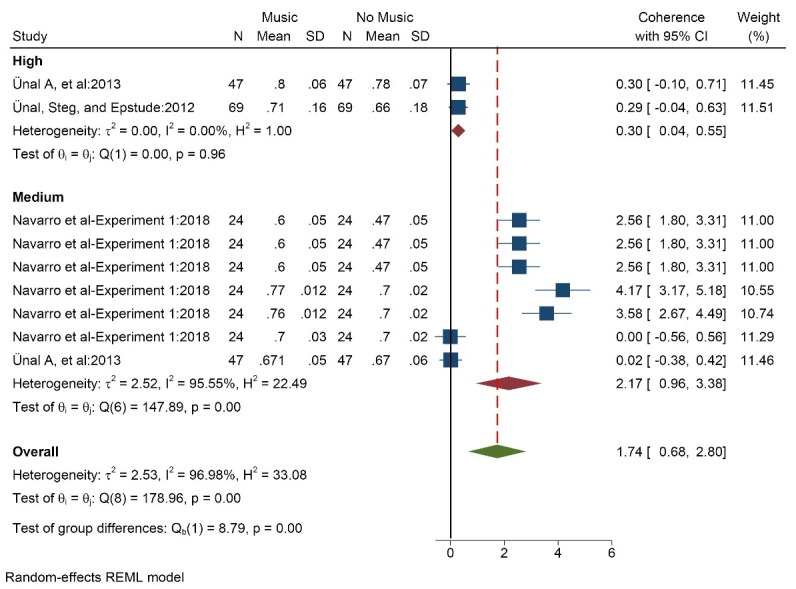
Effect of listening to music on coherence based on the music volume

 Three studies (9 data/conditions) with 140 participants (mean age of 22 years) measured the effect of music on coherence. Music was chosen by the participants. The results showed that music in both high volume (SMD = 0.30 [0.04, 0.55]) and medium volume (SMD = 2.17 [0.96, 3.38]) and totally (SMD = 1.74 [0.68, 2.80]) increased the coherence (Figure 5). The results of the heterogeneity test showed that there is a high degree of heterogeneity among studies (I^2^ = 96.9%). Per Egger’s test there is high probability of publication bias (*P* < 0.001, Z = 7.44) (Supplementary file 3, funnel plot D). The results based on music tempo also showed that music in all three modes—fast, medium, and slow—reduced coherence.

####  Effect of music on other indicators of driving performance

 The results of the study by Hughes et al showed that listening to music had a positive effect on lane change, lane position variability, mean peripheral detection task response time(s), and increased speed variability.^41^ Navarro et al state that listening to music improves the participants’ performance in gaining adjustments relative to the following vehicle, but it shortens the intravehicular time.^42^ The results of the study by Ünal et al also showed that music had a positive effect on the standard deviation of lateral positioning (SDLP) index.^14^ Henry stated that listening to music significantly increased the number of driving errors.^30^ Febriandirza et al showed that listening to classical music improved SDLP and the standard deviation of speed, while hard rock music had a negative effect on these two indicators.^29^ Miao et al stated that while fast and medium-tempo music have a negative effect on drivers’ hazard perception, slow-tempo music has a positive effect on this index.^24^

###  Effect of music on the physiological indicators of drivers

 Data for 30 indicators were extracted from eight experiments that studied the effect of music on the physiological indicators of drivers. Music affected physiological indicators; 16 indicators (53.3%) significantly, while 14 indicators (46.7%) insignificantly. In terms of positive or negative impact, positive impact was reported in 19 indicators (63.3%), and negative impact was reported in 11 indicators (36.7%).

####  Effect of music on heart rate

**Figure 6:**
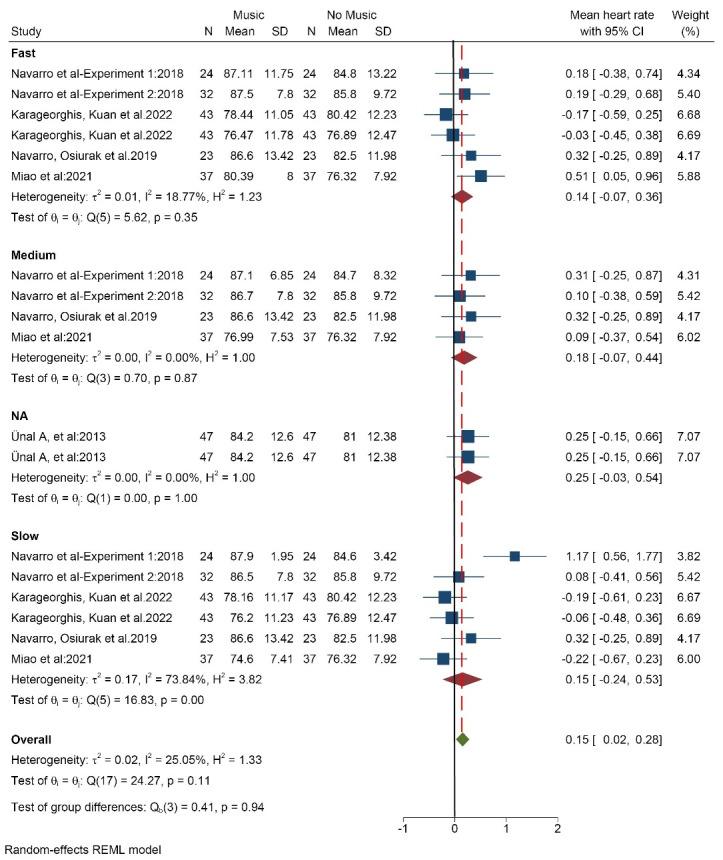
Effect of listening to music on the mean heart rate based on the music volume Effect of music on heart rate variability

 Five studies (18 data/conditions) with 206 participants (mean age of 24.5 years) measured the effect of music on the participants’ heart rate. The results showed that high volume music (SMD = -0.03 [-0.32, 0.26]) decreased the heart rate of drivers. But medium volume (SMD = 0.24 [0.04, 0.44]) and low volume (SMD = 0.12 [-0.10, 0.34]) music increased heart rate. Totally (SMD = 0.15 [0.02, 0.28]), the heart rate was increased by listening to music while driving (Figure 6). The results of the heterogeneity test showed that there is low heterogeneity (I^2^ = 25%). There is high probability of publication bias (Egger test *P* < 0.004, Z = 2.81) (Supplementary file 3, funnel plot E). The results based on music tempo also indicated that music increased the heart rate in all three modes: fast, medium, and slow.

**Figure 7:**
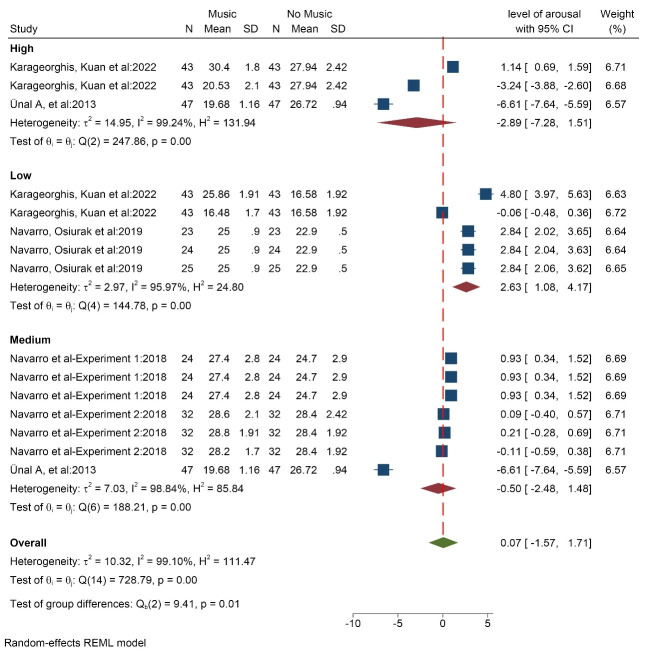
Effect of listening to music on the heart rate variability based on the music volume

 Five studies (13 data/conditions) with 146 participants (mean age 25.6 years) measured the effect of music on the participants’ heart rate variability. The results showed that the heart rate variability score in the group with high-volume music (SMD = -0.23 [-0.46, 0.00]) was lower than the group without music. But in the group with medium-volume music (SMD = 0.02 [-0.39, 0.42]) and low-volume music (SMD = 1.53 [0.25, 2.81]) the average score was higher. In general, the average score was higher in the music group (SMD = 0.45 [-0.20, 1.11]) (Figure 7). The results of the heterogeneity test indicated a considerable degree of heterogeneity (I^2^ = 95.3%). The probability of publication bias was low (Egger test *P* = 0.020, Z = 2.32) (Supplementary file 3, funnel plot F). The analysis results based on music tempo showed that in all three modes—fast, medium, slow, and the average— heart rate variability score was higher in the group with music.

###  Effect of music on the psychological indicators of drivers

 In 10 experiments, the effect of music on the psychological indicators of drivers was indicated. The data for 55 indicators were extracted. Music affected 41 indicators (74.5%) significantly effect, while 14 indicators (25.5%) insignificantly. In terms of positive or negative impact, positive impact was reported in 33 indicators (60%) and negative impact was reported in 22 indicators (40%).

####  Impact of listening to music on the arousal index

**Figure 8:**
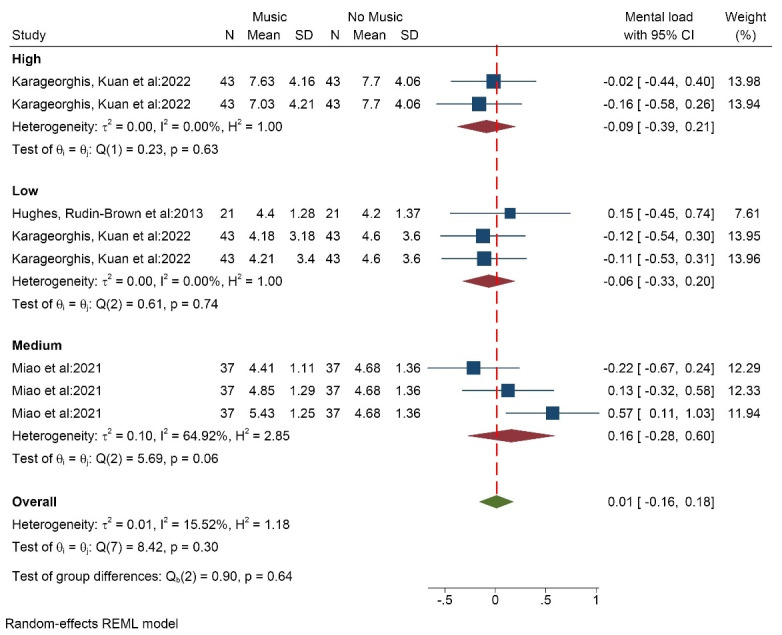
Effect of listening to music on the level of arousal based on the music volume

 Five studies (15 data/conditions) with 169 participants (with an average age of 24.8 years) measured the effect of music on the level of arousal index. The results showed that high volume (SMD = -2.89 [-7.28,1.51]) and medium volume (SMD = -0.50 [-2.48, 1.48]) music reduced the level of arousal. But music with low volume (SMD = 2.63 [1.08, 4.17]) increased the level of arousal. Totally, listening to music while driving led to an increase in the level of arousal (SMD = 0.07 [-1.57, 1.71]) (Figure 8). The results of the heterogeneity test showed that there is a high level heterogeneity (I^2^ = 99.1%). The probability of publication bias was low (Egger test *P* = 0.170, Z = -1.37) (Supplementary file 3, funnel plot G). The results based on music tempo also showed that music increases the level of arousal in all three modes: fast, medium, and slow.

####  Effect of music on the mental load index

**Figure 9:**
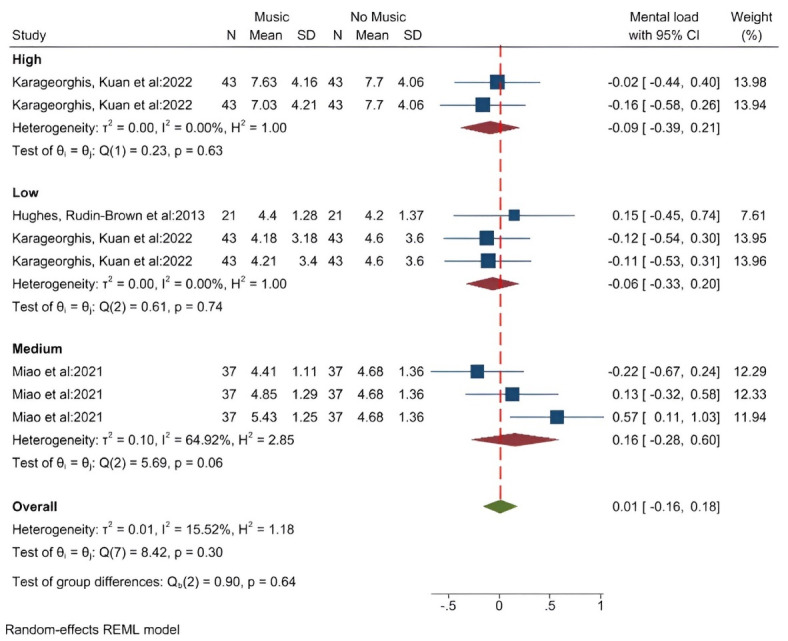
Effect of listening to music on the mental load based on the music volume

 Three studies (8 data/conditions) with 101 participants (average age of 28 years) measured the effect of music on the mental load index using the NASA Task Load Index (NASA-TLX) tool. The results showed that the average score of mental load in the groups with high volume music (SMD = -0.09 [-0.39, 0.21]) and low volume music (SMD = -0.06 [-0.33, 0.20]) was lower than the group without music. But in the group with medium-volume music (SMD = 0.16 [-0.28, 0.60]), the average score of mental load was higher compared to the group without music. In general, listening to music increased the average score of mental load (SMD = 0.01 [-0.16, 0.18]) (Figure 9). The results of the heterogeneity test showed that there is low heterogeneity in the findings of the studies (I^2^ = 15.5%). The results of measuring the publication bias also showed that the probability of publication bias is low (Egger test *P* = 0.293, Z = 1.05) (Supplementary file 3, funnel plot H). The results of the analysis showed that, based on music tempo, in both fast and medium modes, the average mental load score is higher, but in slow mode, it is lower.”

####  Effect of music on other psychological indicators of drivers

 Brodsky and Slor’s study results showed that listening to music has a meritorious impact on improving mood and enjoyment. But music has caused an increase in the possibility of aggressive driving, severe driver miscalculations, and violations.^38^ Navarro et al reported that listening to music led to a significant increase in the mean pleasantness score among drivers.^42^ The results of the study by Karageorghis et al indicated that listening to music, regardless of its volume and tempo, increased the affective valence score among the participants.^43^ Cassidy and Macdonald RA showed that the music chosen by the participants increased the enjoyment index and decreased the distraction index, while the high-arousal music chosen by the researchers had the opposite effect.^28^

###  The results of the quality reporting assessment

 The average assessment score of the reporting quality of the studies was calculated at 35.87 (out of 56). The lowest scores were in “Simulation development” and “Description of simulation feedback or debriefing” items, and the highest scores were in “Results” and “Discussion” items (Supplementary file 4).

## Discussion

 The present systematic review and meta-analysis study assessing the effect of music on driving performance and its physiological and psychological indicators, combine the results from all the available literature in this field, providing valuable insights into this intriguing area of research and revealing some interesting patterns, which will be discussed in detail in this section.

 Most of the studies were conducted in HICs using simulators which may limit the result applicability in low- and middle-income countries (LMICs) due to inaccessibility of simulator technologies. As the results of the current study showed, out of 20 investigated experiments, 17 were conducted using simulators, laboratories, or controlled conditions. According to the World Health Organization, 93% of the deaths caused by traffic accidents in the world occur in LMICs, even though they have about 60% of the world’s vehicles.^1^ Therefore, there is a need for essential supports from international organizations and HICs to transfer the required technologies to conduct such studies in LMICs. This will enhance the capacity and efficiency of these technologies contributing to reduction of traffic accidents in LMICs.

 As mentioned, the results of the study indicated that the use of simulators to measure the impact of music on driving is very common and widely used. Although simulators have many advantages, there may be some limitations.^44,45^ The most important limitation is the absence of real driving feeling as the real driving conditions are not simulated, so the effect of the music cannot be measured correctly. Evaluating the effect of music in real driving conditions can overcome this limitation to some extent, however new technologies cannot be easily applied in real driving conditions. by using simulators and other up-to-date technologies as much as possible in real driving conditions, we can achieve more successes.

 It is noteworthy that cohort and survey studies were excluded as they are different from included controlled studies (simulators, laboratory conditions, and controlled driving on the road), in terms of study design, however the main advantage of these types of studies regardless of their limitations, is the large sample size as analyzing the large scale data results in better understand of the impact of variables at the level of the target population.

 The results showed that high and medium volume music increases the average driving speed, and music with low volume decreases it. It is important to take into account the environment in which these results were acquired, such as any potential safety issues related to music when driving at higher speeds.

 Among the three categories of low, medium, and high volume, the high volume category was the most frequently repeated in the studies. Perhaps one of the main reasons for this is the public belief that high-volume music has many negative effects compared to lower volumes. Many studies showed that high volume has many negative effects on drivers and various driving indicators.^36,40,46^ But in the studies conducted by Ünal et al, the volume of music did not affect the performance of drivers.^14,25^ On the other hand, in some other studies, high-volume music had positive and effective results and improved the performance of drivers. Dibben and Williamson study results showed that high-volume music reduces the fear of drivers and has a positive effect on increasing drivers’ alertness.^8^ Also, the results of the study by Hargreaves et al showed that high-volume music reduces boredom and fatigue caused by driving and thus has a positive effect.^47^ Similar to the results of the study by Millet et al, who reported the effect of music on vehicular performance using a meta-analysis of 12 studies,^16^ the results of the present study also showed that high-volume music has diverse effects on different indicators. It has a negative effect by increasing the average driving speed and average reaction/response time and decreasing heart rate, level of arousal, and mental load. The results showed that although music in every mode reduces the average reaction/response time, it has a positive effect on reducing response delay and increasing coherence. This suggests that music helps speed up drivers’ decision-making, which may be especially helpful in situations that call for quick answers, including avoiding collisions or navigating through traffic jams. Therefore, according to the characteristics and needs of the driver, environment, and driving conditions, recommendations can be made to make listening to music more effective. However, it is important to emphasize the substantial heterogeneity and probable publication bias, which highlights the need for more thorough research in this area.

 Classic rock was the most used type of music in the studies. It seems that compared to the volume and tempo of music, little attention has been paid to the effect of the type of music genre,^48^ so this issue can be one of the interesting research ideas. Although there are many contradictions in the field of the influence of music genre on driving, in general, it is believed that music with a soft genre, such as classical music, has a greater effect on calming drivers and inducing a positive mood, while music with a hard genre, such as rock, has a great impact on inducing dangerous driving and feelings of tension.^49,50^ According to the results of the study of Quotemehappy Insurance in England, drivers who listened to heavy metal music showed more violent behaviors compared to the group that listened to soft music; they observed fewer speed limits and reported more dangerous and near-accident drivings.^51^ In another study conducted in England in 2009, the results showed that 70% of people who were fined for not complying with the speed limit during the last year listened to “pounding fast dance music”.^52^ Other studies reported similar results.^53-55^

 Moving on to physiological indicators, this investigation contends that the effects of music on heart rate and heart rate variability in drivers vary. Music played at medium or low volume increases heart rate, whereas music played at high volume decreases it. Both high and low-loudness music have different effects on heart rate variability, which is an equally intriguing result. These findings demonstrate the intricate interaction between music and driving-related physiological reactions.

 The point that should be mentioned is the influence of culture and social structures. Because the impact of music may be different in different cultural and social contexts, it is recommended to pay attention to the impact of these issues in future research. Also, the impact of demographic variables such as gender, age, and occupation can also be considered by researchers in future studies.

 Although, in recent years, extensive research has been conducted on the different aspects of music and driving, it seems that there is still a need for more research in the future. Moreover, there are some gaps in the literature regarding the safety implications of increased speed and response time associated with music and the role of individual music preferences in driving performance. Therefore, it is highly suggested that future experiments focus on these factors.

 Furthermore, considering the main outcomes such as the number of accidents leading to injury or death, using large sample sizes, providing real driving conditions, paying attention to the impact of background variables such as cultural issues and social structures, and also developing the research projects in underprivileged countries are recommended. It is noteworthy that the authorities and researchers should make efforts to alter the relatively negative public view on the impact of music on driving performance and provide the necessary trainings regarding appropriate music selection based on driving conditions and mentioned factors. Cooperation with music experts for this purpose can be very helpful.

 Although this study provides a comprehensive and transparent review for decision-makers, drivers, and researchers, it has some limitations. There was a great variety in the variables of the included studies, and it was not possible to combine and summarize them regardless of these variables; thus, the number of records included in each subgroup was limited, which can distort the results. Another limitation was that in some studies, due to the way the results were reported, it was not possible to extract information or compare the music group with the non-music group, so meta-analysis could not be fulfilled.

## Conclusion

 This systematic review combines the results of studies conducted in the field of evaluating the effect of music on driving performance. It is stated that in some indicators and situations, listening to music, especially high volume and fast tempo music, has negative effects on driving performance. However music has a positive effect on improving driving safety and physiological and psychological indicators. Hence, it is advisable to identify and employ appropriate music based on the conditions of drivers and driving, while also educating drivers about this selection.

## Acknowledgments

 The authors would like to acknowledge the Road Traffic Injury Research Center of Tabriz University of Medical Sciences and the National Brain Mapping Laboratory (NBML) for helping with data extraction for this study. This study is part of a PhD thesis with registration number 68925 at Tabriz University of Medical Sciences (Tabriz, Iran).

## Competing Interests

 The authors declare no potential conflicts of interest concerning the research, authorship, or publication of this article.

## Ethical Approval

 Ethical approval for the study protocol was obtained from the Medical Ethics Board of Trustees (MEBoT) affiliated with the Tabriz University of Medical Sciences (IR.TBZMED.REC.1400.1050).

## Supplementary Files


Supplementary file 1. Search strategy

Supplementary file 2. Data extraction sheet

Supplementary file 3. Funnel Plots for assessing probability of publication bias

Supplementary file 4. Quality assessment
