# Supplementary material for: Effect of music on driving performance and physiological and psychological indicators: A systematic review and meta-analysis study
Source: Health Promot Perspect. 2023 Dec 16;13(4):267–79. doi: 10.34172/hpp.2023.32 (PMC10790125; doi:10.34172/hpp.2023.32)
Supplement: Supplementary file 1 — Search strategy [file hpp-13-267-s001.pdf]

Ghojazadeh et al, **Health Promotion Perspectives**, 2023, 13(4), 267-S1.
doi: 10.34172/hpp.2023.32
https://hpp.tbzmed.ac.ir

Supplementary file 1. Search Strategy

PubMed Search Strategy

Set	Strategy	Results
#1	"Accidents, Traffic"[Mesh] OR (("Motor Vehicles"[Mesh:NoExp] OR driv* OR "Automobiles"[Mesh] OR "Motorcycles"[Mesh] OR traffic[tiab] OR vehicle[tiab] OR vehicular[tiab] OR car[tiab] OR cars[tiab] OR automobile[tiab] OR automobiles[tiab] OR motorcycle[tiab] OR motorcycles[tiab] OR taxi[tiab] OR cab[tiab] OR road[tiab] OR pedestrian[tiab] OR pedestrians[tiab]) AND (accident[tiab] OR accidents[tiab] OR injury[tiab] OR injuries[tiab] OR "Wounds and Injuries"[Mesh] OR "injuries" [Subheading]))	**113,383**
#2	((((music*[Title/Abstract]) OR (Symphony[Title/Abstract])) OR (Rhythm*[Title/Abstract])) OR (Orchestra[Title/Abstract])) OR (Song[Title/Abstract])	**171,464**
#3	**#1 AND #2**	**443**

Scopus Search Strategy

Set #		Results
1	TITLE-ABS-KEY ((("Motor Vehicles" OR automobiles OR motorcycles OR traffic OR vehicle OR vehicular OR car OR cars OR automobile OR motorcycle OR taxi OR cab OR road OR pedestrian OR pedestrians) AND (accident OR accidents OR injury OR injuries))) AND (LIMIT-TO (SUBJAREA , "MEDI") OR LIMIT-TO (SUBJAREA , "ENGI") OR LIMIT-TO (SUBJAREA , "SOCI") OR LIMIT-TO (SUBJAREA , "HEAL") OR LIMIT-TO (SUBJAREA , "NURS") OR LIMIT-TO (SUBJAREA , "ECON")) AND (LIMIT-TO (DOCTYPE , "ar") OR LIMIT-TO (DOCTYPE , "re")) AND (LIMIT-TO (LANGUAGE , "English"))	**108,643**

**2**	(TITLE-ABS-KEY (music*) OR TITLE-ABS-KEY (symphony) OR TITLE-ABS-KEY (rhythm) OR TITLE-ABS-KEY (orchestra) OR TITLE-ABS-KEY (song))	**531,052**
**3**	#1 AND #2	**675**

Search Strategy Web of Science

**Set #**		**Results**
**1**	(TI=("Motor Vehicles") OR TI=(automobiles) OR TI=(motorcycles) OR TI=(traffic) OR TI=(vehicle) OR TI=(vehicular) OR TI=(car) OR TS=(cars) OR TI=(automobile) OR TI=(motorcycle) OR TI=(taxi) OR TS=(cab) OR TI=(road) OR TI=(pedestrian) OR TI=(pedestrians) OR TI=(accident*) OR TI=(injur*)) AND (DT==("ARTICLE") AND LA==("ENGLISH"))	**594,638**
**2**	(AB=(music*) OR AB=(symphony) OR AB=(rhythm) OR AB=(orchestra) OR AB=(song)) AND (DT==("ARTICLE") AND LA==("ENGLISH"))	**160,945**
**3**	#1 AND #2	**1,532**
