# Supplementary material for: Effect of music on driving performance and physiological and psychological indicators: A systematic review and meta-analysis study
Source: Health Promot Perspect. 2023 Dec 16;13(4):267–79. doi: 10.34172/hpp.2023.32 (PMC10790125; doi:10.34172/hpp.2023.32)
Supplement: Supplementary file 2 — Data extraction sheet [file hpp-13-267-s002.pdf]

Ghojazadeh et al, **Health Promotion Perspectives,** 2023, 13(4), 267-S2.
doi: 10.34172/hpp.2023.32
https://hpp.tbzmed.ac.ir

Supplementary file 2. Data extraction sheet

	Authors	Year	Country	participants						procedure			
				Partici pants numbe r	Particip ants age (MN/M D)	Parti cipan ts male	Participa nts female	Drivin g experie nce (Year)	listen to music (%)	Test drive (KM)	Experi mental conditi ons	familia risatio n drive	testing time
1	G. M. Hughes et al[1]	2013	**Australia**	21	35	1.00	20.00	NA	NA	6.6	S	Yes	daylight
2	W. Brodsky et al[2]	2013	pied pale	85	17.6	49	36	0.7	86	39.4	R	Yes	NA
3	J. Navarro et al[3]	2018	France	24	22.7	13	11	4.2	NA	NA	s	Yes	NA
4	J. Navarro et al[3]	2018	France	32	23	20	12	6	NA	NA	s	Yes	NA

5	W. Consiglio et al[4]	2003	USA	22	21	11	11	NA	NA	NA	L	Yes	NA
6	C. I. Karageorghis et al[5]	2022	UK	43	29.8	23	23	10.4	NA	NA	s	Yes	NA
7	H. C. Beh et al[6]	1999	Australia	45	20.4	21	24	least 2	NA	NA	L	Yes	NA
8	J. Navarro et al[7]	2019	France	23	NA	NA	NA	NA	NA	NA	S	Yes	NA

9	A. B. Ünal et al[8]	2013	Netherla	47	20.7	26	21	2.6	NA	NA	S	Yes	daylight	
10	D. B. Bellinger et al[9]	2009	USA	27	20.9	16	11	5.3	96	NA	L	Yes	NA	
11	A. Mohunta[10]	2022	India	8	45	4	4	NA	NA	NA	R	NA	daylight	
12	Z. N. Jimison[11]	2014	USA	165	21	47	118	NA	NA	NA	S	Yes	NA	
13	E. L. Henry[12]	2006	USA	36	22.4	10	26	6.4	94	NA	L	Yes	NA	
14	A. Febriandirza et al[13]	2017	China	98	24.4	49	49	5.3	100	NA	S	Yes	NA	
15	G. G. Cassidy et al[14]	2010	UK	70	20.5	38	32	NA	NA	NA	S	Yes	NA	

16	G. Cassidy et al[15]	2009	UK	125	21.2	60	65	NA	NA	NA	S	Yes	NA
17	W. Brodsky[16]	2001	pied pale	28	25	10	18	7	100	NA	S	Yes	NA
18	M. A. Alves[17]	2019	Brazil	5	20.8	0	5	NA	NA	3	R	NA	daylight
19	A. B. Ünal et al[18]	2012	Netherla	69	21	23	46	2.92	NA	NA	S	Yes	NA
20	L. Miao et al[19]	2021	China	37	23.5	23	14	0.62	93.2	NA	S	Yes	daylight

		simulator								Music
**control**	**fixed or protable**	**open source**	**screen (Inch)**	**screen view**	**music numb**	**Music/test duration**	**music**	**Music**	**Music**	**Music**
-Faros (Lanni	Portable	NA	NA	120	2	NA	NA	Rsel	yes	NA
					12	NA no		Dsel	yes	NA
NO simulator	NO simulato	O simulat	NO simulator	NO simulator						
8						34	no	Rsel	no	M
Logitech G27®	fixed	yes	19	NA	7	55	NA	Dsel	yes	S, M, F
Logitech G27®	fixed	yes	20	NA	12	30	no	Rsel	yes	S, M, F

NO simulator	NO simulato	O simulat	NO simulator	NO simulator	1	NA	NA	Rsel	NA	NA
OpenDS 4.0	fixed	yes	32	NA	4	16	NA	Rsel	yes	F, S
NO simulator	NO simulato	O simulat	14	NA	NA	20	NA	Rsel	NA	NA
BB_Sim	NA	yes	48	137.5	NA	50	N	Dsel	NA	S, M, F

				240	NA	30	NA	Dsel	yes	NA
StSoftware	fixed	NA	NA							
NO simulator	NO simulator	O simulat	NO simulator	NO simulator	1	NA	NA	Rsel	yes	NA
NO simulator	NO simulator	O simulat	NO simulator	NO simulator	2	NA	NA	Rsel	yes	NA
IM (Build 2.06.00)		NA	NA	NA	10	24	N	Rsel, Dsel	yes , No	S, M, F
NO simulator	NO simulator	O simulat	NO simulator	NO simulator	1	NA	NA	Rsel	yes	NA
NA	fixed	NA	NA	180	NA	35	NA	Rsel	NA	S, M, F
NA	NA	NA	NA	NA	NA	NA	NA	Rsel, Dsel	no	S, M, F

server Pack	NA	NA	NA	NA	25	NA	NA	Dsel, Rsel	yes	S, M, F
Desk pro	NA	NA	17	NA	12	90	NA	Rsel	NA	S, M, F
NO simulator	NO simulator	O simulat	NO simulator	NO simulator	5	20	N	Rsel	NA	NA
NA	Fixed	NA	NA	180	NA	35	NA	Dsel	yes	NA
NA	NA	NA	NA	NA	3	30	NA	Rsel	yes	S, M, F

Music [illegible]	Music [illegible]	Music [illegible]	Outcome name	Outcome in [illegible]
			Mean speed	54.8
			Speed variability	4.28
L	yes	NA	Lane position variability	0.27
			Mean lane excursions	0.14
			Mean PDT response time(s)	1.15
			Average mental workload (out of 10)	4.4
			Participant mood states (POMS)	1.89
			Effect of music on mood	0.72
H	NA	Rk, Ea, El, TV, HR, Rp, RB,	at least one violation (%)	98
			Severity of deficient driver behavior	53.89
			event-frequency	11.93
			event-severity	189
			Participant mood states (POMS)	1.24
			Effect of music on mood	0.06
			at least one violation (%)	90
H	no	Ea, Jz, O	Severity of deficient driver behavior	40.42
			event-frequency	10.09(6.8)
			event-severity	157(111)
			Mean heart rate	87,9
			mean level of arousal (12 to 48)	27.4
			mean level of pleasantness (16 to 64)	51.4
			Mean intervehicular time	3.8
M	NA	NA	Coherence	0.6
			Delay (in seconds)	3.03
			Gain	0.54
			Mean heart rate (fast tempo)	87.5
			Mean heart rate (medium tempo)	86.7
			Mean heart rate (slow tempo)	86.5
			mean level of arousal (12 to 48) (fast tempo)	28.6
			mean level of arousal (12 to 48) (medium tempo)	28.8
			mean level of arousal (12 to 48)(slow tempo)	28.2
			mean level of pleasantness (16 to 64)(fast tempo)	50.9
			mean level of pleasantness (16 to 64)(medium tempo)	50.5
			mean level of pleasantness (16 to 64)(slow tempo)	50
			Mean intervehicular time (Slow)	3.7
			Mean intervehicular time (medium)	3.6
M	NA	Rk, Co, Jz, RB	Mean intervehicular time (Fast)	3.7
			Coherence (Slow)	0.77
			Coherence (medium)	0.76
			Coherence (Fast)	0.7
			Delay (in seconds) (Slow)	2.5
			Delay (in seconds) (medium)	2.6

Delay (in seconds) (Fast)				2.5
Gain				0.77
Gain				0.74
Gain				0.66
NA	NA	Rk	Mean reaction time	0.408
NASA TaskLoadIndex(NASA-TLX) (H-F)				7.63
NASA TaskLoadIndex(NASA-TLX) (H-S)				7.03
NASA TaskLoadIndex(NASA-TLX) (L-F)				4.18
NASA TaskLoadIndex(NASA-TLX) (L-S)				4.21
Affective arousal (H-F)				5.7
Affective arousal (H-S)				3.85
Affective arousal (L-F)				4.85
Affective arousal (L-S)				3.09
L,H	yes	con	Affective valence (H-F)	6.8
			Affective valence(H-S)	7.02
			Affective valence (L-F)	7.13
			Affective valence (L-S)	7.28
			Mean heart rate (H-F)	78.44
			Mean heart rate (H-S)	78.16
			Mean heart rate (L-F)	76.47
			Mean heart rate (L-S)	76.2
			Heart rate variability (RMSSD) (H-F)	38.77
			Heart rate variability (RMSSD) (H-S)	44.89
			Heart rate variability (RMSSD) (L-F)	41.82
			Heart rate variability (RMSSD) (L-S)	49.2
			Mean response time (Stop-light task)/low-intensity music (LIM)/low	392
			Mean response time (Stop-light task)/low-intensity music (LIM)/High	426
			Mean response time (Stop-light task)/High-intensity music (LIM)/low	502.6
L,H	NA	HR	Mean response time (Stop-light task)/High-intensity music (LIM)/High	518
			Mean response time (vigilance task)/low-intensity music (LIM)/low	595
			Mean response time (vigilance task)/low-intensity music (LIM)/High	632
			Mean response time (vigilance task)/high-intensity music (HIM)/low	550
			Mean response time (vigilance task)/High-intensity music (HIM)/high	512.6
			pleasant-unpleasant	50.5
			positive-tired	19.9
			arousal-calm	25
			negative-relaxed	9.1
L	NA	NA		
		Mean heart rate		86.6
		Heart rate variability		5.8
		Mean intervehicular time		4.7
		Mean inter-vehicular time standard deviation.		1.32
		coherence (high)/mean		0.8
		coherence (Moderate)mean		0.671

			delay in response (high)	3.25
			delay in response (Moderate)	3.95
M or H	NA	NA	SDLP (high)/mean	0.246
			SDLP (Moderate)/mean	0.243
			mean level of arousal	3.69
			mean heart rate	84.2
			heart rate variability	6.9
			Response time (moderate volume)	585.6
			Response time (high volume)	584.9
M, H	NA	RK	Reaction time (moderate volume)	389.6
			Reaction time (high volume)	389.9
			Movement time (moderate volume)	196
			Movement time (high volume)	195
L,H	yes	TV	Mean speed (low volume)	43.8
			Mean speed (high volume)	56.6
M	NA	Rk	Mean speed	35.34
L,H	NA	Rk	Mean Error rate (low volume)	17.47
			Mean Error rate (high volume)	16.61
			SDS (Classical Music)	2.08
			SDS (Hard Rock Music)	2.43
			SDLP (Classical Music)	21.25
			SDLP (Hard Rock Music)	22.8
			experience ratings/Control(Classical Music)	6.51
H	NA	Cl, HR	experience ratings/Control (Hard Rock Music)	3.09
			experience ratings/Concentration(Classical Music)	6.68
			experience ratings/Concentration(Hard Rock Music)	3.27
			experience ratings/Enjoyment(Classical Music)	6.59
			experience ratings/Enjoyment(Hard Rock Music)	3.38
			experience ratings/Distraction(Classical Music)	4.45
			experience ratings/Distraction(Hard Rock Music)	8.27
			Lap time (s)/ low-arousal music (70 bpm)	124.1
			Lap time (s)/ low-arousal music (130 bpm)	121.5
			Lap time (s)/ high-arousal music (70 bpm)	101.6
			Lap time (s)/high-arousal music (130pm)	98.8
			Lap time (s)/ self-selected music	99.2
			Inaccuracy/ low-arousal music (70 bpm)	7.3
			Inaccuracy/ low-arousal music (130 bpm)	7.4
			Inaccuracy/ high-arousal music (70 bpm)	15.4
			Inaccuracy/ high-arousal music (130pm)	18.7
			Inaccuracy/ self-selected music	3
			Lap speed (mph)/low-arousal music (70 bpm)	25.1
			Lap speed (mph)/low-arousal music (130 bpm)	27.5
L	NA	Rk, Cl, HR, Jz	Lap speed (mph)/high-arousal music (70 bpm)	40.3
			Lap speed (mph)/high-arousal music (130pm)	43.6
			Lap speed (mph)/self-selected music	43.5
			Distraction/ low-arousal music (70 bpm)	4
			Distraction/ low-arousal music (130 bpm)	3.8

			Distraction/ high-arousal music (70 bpm)	5.9
			Distraction/high-arousal music (130pm)	7.6
			Distraction/ self-selected music	2.1
			Enjoyment/ low-arousal music (70 bpm)	3.6
			Enjoyment/ low-arousal music (130 bpm)	3.6
			Enjoyment/ high-arousal music (70 bpm)	2.2
			Enjoyment/high-arousal music (130pm)	2.4
			Enjoyment/ self-selected music	9.6
			Lap time (s)/ low-arousal music (70 bpm)	116.2
			Lap time (s)/high-arousal music (130pm)	94.3
			Lap time (s)/ self-selected music	93.1
			Inaccuracy/ low-arousal music (70 bpm)	9.9
L	yes	Rk	Inaccuracy/ high-arousal music (130pm)	21.2
			Inaccuracy/ self-selected music	5.4
			Lap speed (mph)/low-arousal music (70 bpm)	23.6
			Lap speed (mph)/high-arousal music (130pm)	38.3
			Lap speed (mph)/self-selected music	41.5
			Heart rate variability	2.86
			Mean speed (slow tempo)	141.1
H	yes	Rk, Co, Jz, RB	Mean speed (medium-tempo)	143.1
			Mean speed (fast tempo)	147.4
NA	NA	NA	Heart rate variability (linear analysis-RMSSD)	29.72
coherence				0.71
delay in response				3.44
H	NA	NA	SDS	6.72
			time-to-contact with the parked car driving off	1.12
			Subjective Load Value (slow tempo)	4.41
			Subjective Load Value (medium-tempo)	4.85
			Subjective Load Value (fast tempo)	5.43
			Heart Rate (slow tempo)	74.6
			Heart Rate (medium-tempo)	76.99
			Heart Rate (fast tempo)	80.39
			R-R Interval (ms) (slow tempo)	816.7
			R-R Interval (ms) (medium-tempo)	791
			R-R Interval (ms) (fast tempo)	777.4
M	no	light melodies		
			Skin Temperature (slow tempo)	32.6
			Skin Temperature (medium-tempo)	33.1
			Skin Temperature (fast tempo)	33.3
			Respiratory Rate (brpm) (slow tempo)	17.8
			Respiratory Rate (brpm) (medium-tempo)	17.88
			Respiratory Rate (brpm) (fast tempo)	18.15
			Hazard Perception (slow tempo)	31.53
			Hazard Perception (medium-tempo)	26
			Hazard Perception (fast tempo)	25.86

SD/SE	Outcome in	SD/SE	Outcome	
		Outcome direction		
0.9	60.25	1.22	Y	Po
0.22	4	0.13	N	Ne
0.01	0.32	0.02	Y	Po
0.01	0.22	0.03	y	Po
0.03	1.05	0.03	y	Ne
0.28	4.2	0.3	N	Po
0.59	1.17	0.89	y	Po
0.88			y	Po
	92		y	Ne
31.4	50.68	37.3	y	Ne
7.5	10.12	6.8	y	Ne
132	158	120	y	Ne
0.77	1.17	0.89	y	po
0.83			y	Po
	92		y	Ne
25.8	50.68	37.3	y	Po
	10.12 (6.8)		y	Po
	158 (120)		y	Po
0.4	84,6	0.7	y	Ne
2.8	24.7	2.9	y	Po
3	48.6	2.9	y	po
0.1	4.5	0.2	y	Ne
0.05	0.47	0.05	N	po
0.21	3.48	0.3	N	po
0.04	0.42	0.04	N	po
	85.8		N	Ne
	85.8		N	Ne
	85.8		N	Ne
	28.4		N	Po
	28.4		N	Po
	28.4		N	Ne
	50.3		N	Po
	50.3		N	Po
	50.3		N	Ne
0.12	4	0.1	N	Ne
0.11	4	0.1	N	Ne
0.12	4	0.1	N	Ne
0.012	0.7	0.02	y	Po
0.012	0.7	0.02	y	Po
0,030	0.7	0.02	y	Po
0.12	3	0.25	N	Po
0.11	3	0.25	N	Po

Po Po Po Po Ne

Ne Ne Ne Ne Po Ne Po

Ne

Po Po Po Po po po po po Ne Po Ne Po Po Po Po Po Po Po Po Po Po Po

Po Po Ne Po Ne Po Po Po

0.1	3	0.25	N
0.012	0.68	0.012	y
0.013	0.68	0.012	y
0.012	0.68	0.012	y
0.03	0.392	0.03	N
4.16	7.7	4.06	N
4.21	7.7	4.06	N
3.18	4.6	3.6	N
3.4	4.6	3.6	N
1.8	5.24	2.42	N
2.1	5.24	2.42	N
1.91	3.11	1.92	y
1.7	3.11	1.92	y
1.73	4.37	1.88	y
1.61	4.37	1.88	y
1.13	5.17	1.54	y
1.73	5.17	1.54	y
11.05	80.42	12.23	N
11.17	80.42	12.23	N
11.78	76.89	12.47	N
11.23	76.89	12.47	N
28.1	44.49	30.73	N
33.01	44.49	30.73	N
33.99	47.25	38.54	N
39.82	47.25	38.54	N
8	448	10	y
11	448	10	y
10	571	10	y
9	571	10	y
12	654	10.3	y
12.3	655	10.3	y
9	622.6	13	y
11	622.6	13	y
0.6	48.6	1.1	y
0.5	17.8	0.5	y
0.9	22.9	0.5	y
0.9	8.6	0.3	N
2.8	82.5	2.5	Y
0.3	5	0.3	N
0.3	5.1	0.3	y
0.05	1.33	0.06	N
0.06	0.78	0.07	N
0.05	0.67	0.06	N

0.53	3.3	0.51	N	Ne
0.58	5.2	0.48	Y	Ne
0.073	0.256	0.07	N	Po
0.056	0.276	0.07	N	Po
1.16	5.01	0.94	Y	Po
12.6	81	12.38	Y	Ne
0.62	6.89	0.68	N	po
98	601.6	112	N	Ne
84.6	601.6	112	N	Ne
48	387	50.5	N	Po
56.2	387	50.5	N	Po
41.8	214.6	39.3	N	Ne
28.4	214.6	39.3	N	Ne
7.9	53.5	4.87	Y	Po
10.9	53.5	4.87	N	Ne
1.28	34.99	1.33	Y	Ne
3.3	17.5	2.2	Y	Ne
2.58	17.5	2.2	Y	Ne
0.14	2.25	0.11	Y	Po
0.14	2.25	0.11	Y	Ne
0.56	21.75	0.53	Y	Po
0.79	21.75	0.53	Y	Ne
0.5	4.24	0.66	Y	Po
0.7	4.24	0.66	Y	Ne
0.62	4.34	0.73	Y	Po
0.68	4.34	0.73	Y	Ne
0.59	4.32	0.65	Y	Po
0,62	4.32	0.65	Y	Ne
0.52	5.04	0.59	Y	Po
0.65	5.04	0.59	Y	Ne
5.5	112.7	6.1	Y	po
5.6	112.7	6.1	Y	po
5	112.7	6.1	Y	Ne
6.5	112.7	6.1	Y	Ne
7.4	112.7	6.1	Y	Ne
1.9	6.5	2.2	Y	Ne
1.9	6.5	2.2	Y	Ne
2.6	6.5	2.2	Y	Ne
2.9	6.5	2.2	Y	Ne
1.9	6.5	2.2	Y	po
4.4	31.9	3.4	Y	po
3.9	31.9	3.4	Y	po
4.3	31.9	3.4	Y	Ne
4.4	31.9	3.4	Y	Ne
4.5	31.9	3.4	Y	Ne
1.1	3.3	1.1	Y	Ne
1	3.3	1.1	Y	Ne

1.2	3.3	1.1		Y	Ne
1.1	3.3	1.1		Y	Ne
0.9	3.3	1.1		Y	po
1.2	2.7	1.2		Y	po
1.2	2.7	1.2		Y	po
2.2	2.7	1.2		Y	Ne
1	2.7	1.2		Y	Ne
1.5	2.7	1.2		Y	po
7.7	105.7	9.4		Y	po
7.3	105.7	9.4		Y	Ne
9.8	105.7	9.4		Y	Ne
2.7	9	3		Y	po
5.1	9	3		Y	po
2.1	9	3		Y	Ne
3.2	28.2	3.6		Y	po
7.1	28.2	3.6		Y	Ne
4.7	28.2	3.6		Y	Ne
0.71	3.43	1.3		Y	po
32.1	144.5	30.18	NA		Po
26.9	144.5	30.18	NA		Po
30.9	144.5	30.18	NA		Ne
17.7	26.66	5.4		N	Po
0.16	0.66	0.18		N	Ne
1.29	4.65	1.92		Y	Po
1.48	5.74	1.81		y	Ne
0.25	0.97	0.3		N	Po
1.11	4.68	1.36		y	Ne
1.29	4.68	1.36		y	Po
1.25	4.68	1.36		y	Po
7.41	76.32	7.92		y	Ne
7.53	76.32	7.92		y	Po
8	76.32	7.92		y	Po
91.7	795.5	89.5		y	Ne
85.1	795.5	89.5		y	Po
96	795.5	89.5		y	Po
1	32.8	1		y	Ne
1.1	32.8	1		y	Po
0.9	32.8	1		y	Po
0.64	17.85	0.56		y	Ne
0.55	17.85	0.56		y	Po
0.26	17.85	0.56		y	Po
5.27	28.78	6.96		y	Po
7.18	28.78	6.96		y	Ne
6.13	28.78	6.96		y	Ne

1. Hughes GM, Rudin-Brown CM, Young KL. A simulator study of the effects of singing on driving performance. *Accid Anal Prev.* 2013; 50: 787-92. doi:10.1016/j.aap.2012.07.001
2. Brodsky W, Slor Z. Background music as a risk factor for distraction among young-novice drivers. *Accid Anal Prev.* 2013; 59: 382-93. doi:10.1016/j.aap.2013.06.022
3. Navarro J, Osiurak F, Reynaud E. Does the Tempo of Music Impact Human Behavior Behind the Wheel? *Hum Factors.* 2018; 60(4): 556-74. doi:10.1177/0018720818760901
4. Consiglio W, Driscoll P, Witte M, Berg WP. Effect of cellular telephone conversations and other potential interference on reaction time in a braking response. *Accident Analysis and Prevention.* 2003; 35(4): 495-500. doi:10.1016/S0001-4575(02)00027-1
5. Karageorghis CI, Payre W, Howard LW, Kuan G, Mouchlianitis E, Reed N*, et al.* Influence of music on driver psychology and safety-relevant behaviours: a multi-study inductive content analysis. *TheoreTical issues in ergonomics science.* 2022; 23(6): 643-62. doi:https://doi.org/10.1080/1463922X.2021.2009933
6. Beh HC, Hirst R. Performance on driving-related tasks during music. *Ergonomics.* 1999; 42(8): 1087-98. doi:10.1080/001401399185153
7. Navarro J, Osiurak F, Gaujoux V, Ouimet MC, Reynaud E. Driving Under the Influence: How Music Listening Affects Driving Behaviors. *J Vis Exp.* 2019; (145). doi:10.3791/58342
8. Ünal AB, de Waard D, Epstude K, Steg L. Driving with music: Effects on arousal and performance. *Transportation Research Part F: Traffic Psychology and Behaviour.* 2013; 21: 52-65. doi:https://doi.org/10.1016/j.trf.2013.09.004
9. Bellinger DB, Budde BM, Machida M, Richardson GB, Berg WP. The effect of cellular telephone conversation and music listening on response time in braking. *Transportation Research Part F: Traffic Psychology and Behaviour.* 2009; 12(6): 441-51. doi:10.1016/j.trf.2009.08.007
10. Mohunta A. The Effect of Music Energy on Driving Speed. *The National High School Journal of Science.* 2022: 1-4.
11. Jimison ZN. The effect of music familiarity on Driving: A simulated study of the impact of music familiarity under different driving conditions. 2014.
12. Henry EL. Effect of Music Volume on Simulated Interstate Driving Skills. 2006.
13. Febriandirza A, Chaozhong W, Zhong M, Hu Z, Zhang H. The Effect of Natural Sounds and Music on Driving Performance and Physiological. *Engineering letters.* 2017 25(4).
14. Cassidy GG, Macdonald RA. The effects of music on time perception and performance of a driving game. *Scand J Psychol.* 2010; 51(6): 455-64. doi:10.1111/j.1467-9450.2010.00830.x
15. Cassidy G, Macdonald R. The effects of music choice on task performance: A study of the impact of self-selected and experimenter-selected music on driving game performance and experience. *Musicae Scientiae.* 2009; 13(2): 357-86. doi:10.1177/102986490901300207
16. Brodsky W. The effects of music tempo on simulated driving performance and vehicular control. *Transportation Research Part F: Traffic Psychology and Behaviour.* 2001; 4(4): 219-41. doi:10.1016/S1369-8478(01)00025-0
17. Alves MA, Garner DM, do Amaral JAT, Oliveira FR, Valenti VE. The effects of musical auditory stimulation on heart rate autonomic responses to driving: A prospective randomized case-control pilot study. *Complement Ther Med.* 2019; 46: 158-64. doi:10.1016/j.ctim.2019.08.006
18. Ünal AB, Steg L, Epstude K. The influence of music on mental effort and driving performance. *Accid Anal Prev.* 2012; 48: 271-8. doi:10.1016/j.aap.2012.01.022
19. Miao L, Gu Y, He L, Wang H, Schwebel DC, Shen Y. The influence of music tempo on mental load and hazard perception of novice drivers. *Accid Anal Prev.* 2021; 157: 106168. doi:10.1016/j.aap.2021.106168
