# Supplementary material for: Effect of music on driving performance and physiological and psychological indicators: A systematic review and meta-analysis study
Source: Health Promot Perspect. 2023 Dec 16;13(4):267–79. doi: 10.34172/hpp.2023.32 (PMC10790125; doi:10.34172/hpp.2023.32)

Ghojazadeh et al, **Health Promotion Perspectives,** 2023, 13(4), 267-S3.
doi: 10.34172/hpp.2023.32
https://hpp.tbzmed.ac.ir

Supplementary file 3. Funnel Plots for assessing probability of publication bias

A- Funnel plot for mean of speed

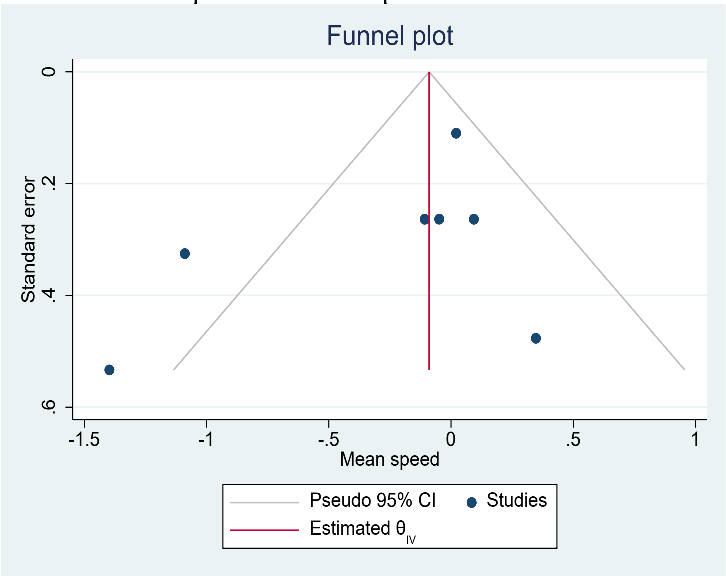

B- Funnel plot for reaction/response time

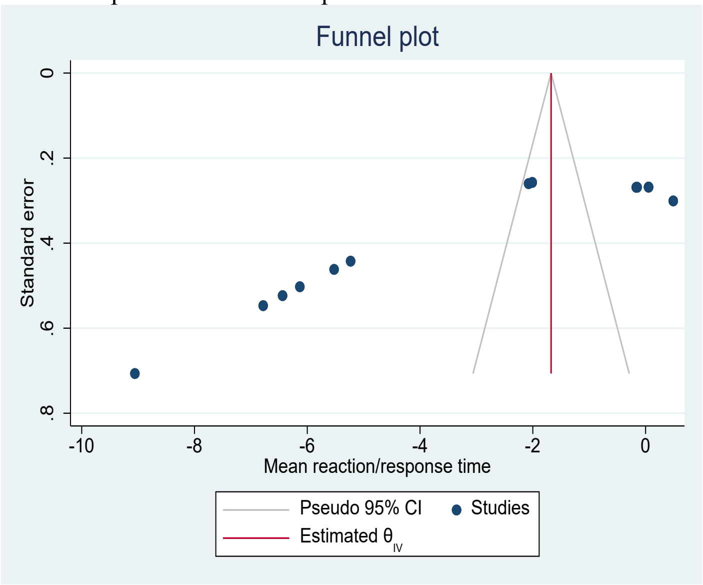

C- Funnel plot for delay in response

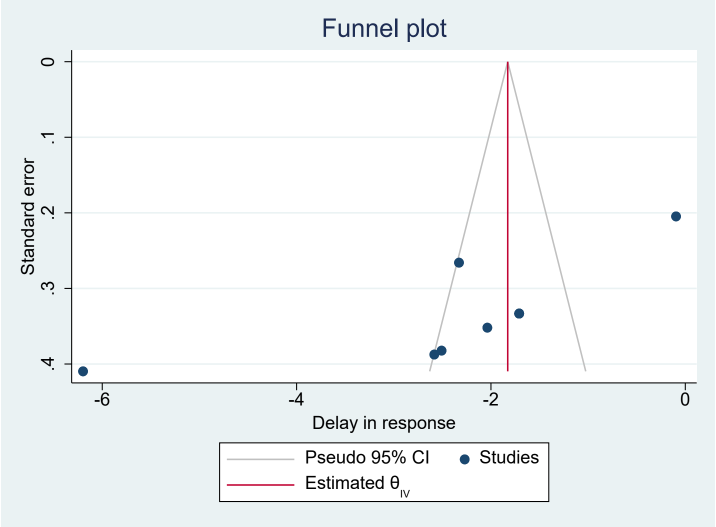

D- Funnel plot for Coherence

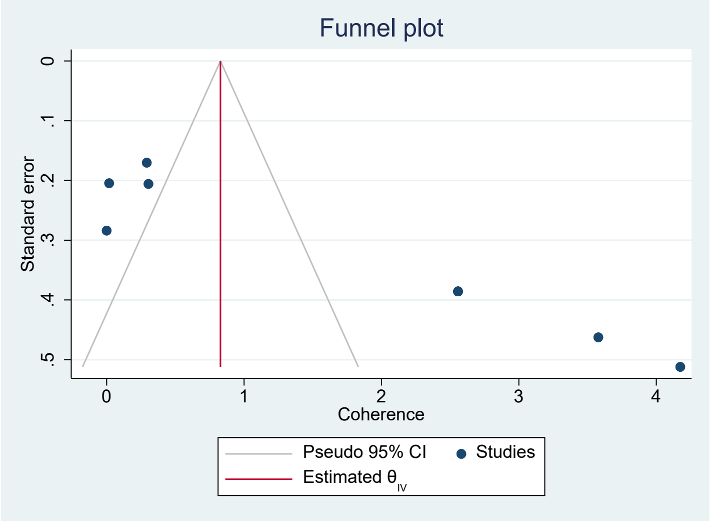

E- Funnel plot for Heart Rate

F- Funnel plot for Heart rate variability

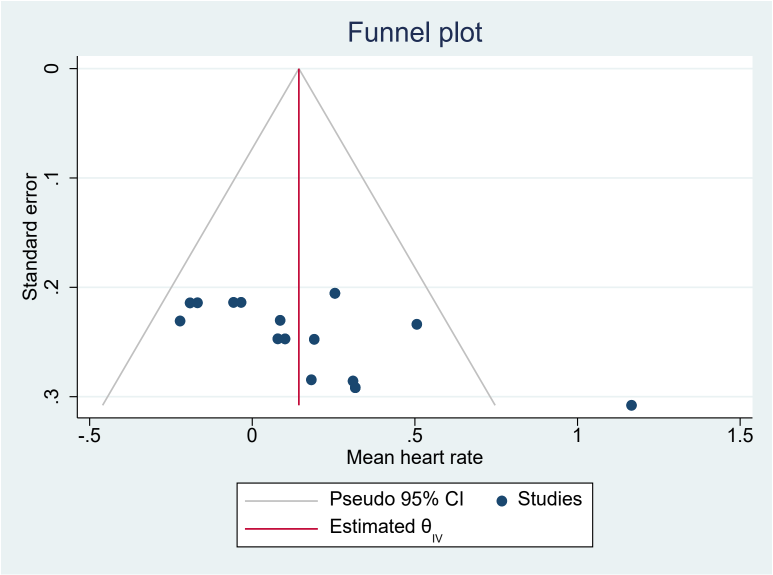

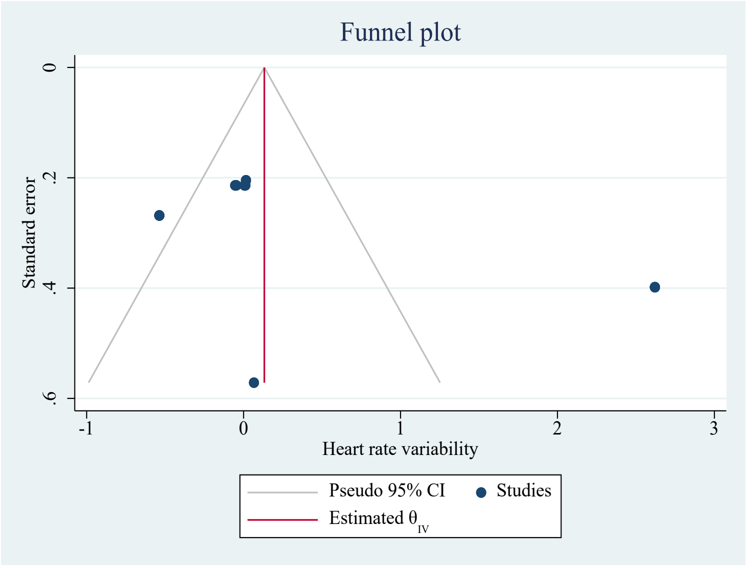

G- Funnel plot for Mean level of arousal

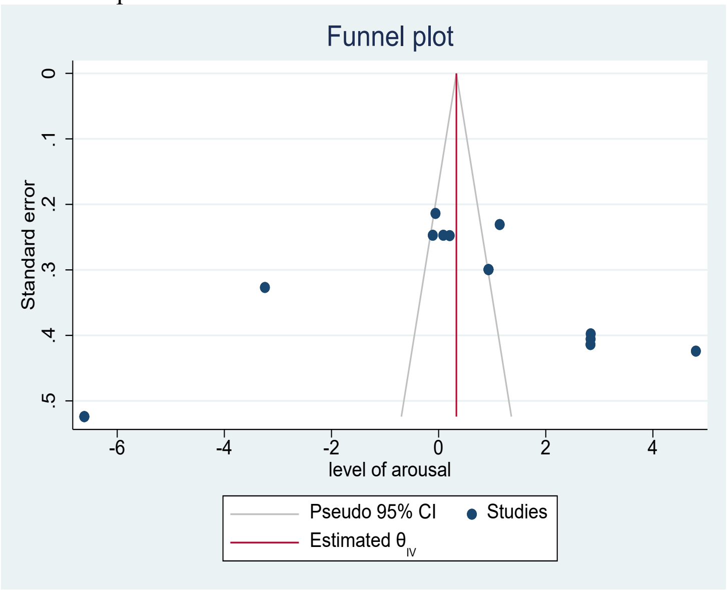

H- Funnel plot for Mental Load

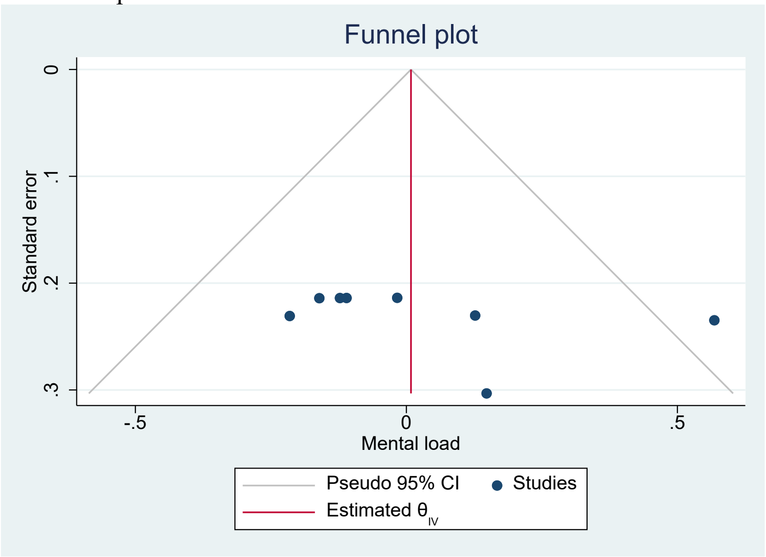

Supplement: Supplementary file 3 — Funnel Plots for assessing probability of publication bias [file hpp-13-267-s003.pdf]
