# Supplementary material for: Effect of music on driving performance and physiological and psychological indicators: A systematic review and meta-analysis study
Source: Health Promot Perspect. 2023 Dec 16;13(4):267–79. doi: 10.34172/hpp.2023.32 (PMC10790125; doi:10.34172/hpp.2023.32)
Supplement: Supplementary file 4 — Quality assessment [file hpp-13-267-s004.pdf]

Ghojazadeh et al, **Health Promotion Perspectives,** 2023, 13(4), 267-S4.
doi: 10.34172/hpp.2023.32
https://hpp.tbzmed.ac.ir

Supplementary file 4. Quality assessment

Author	introduction/background/Rationale	Literature Review	Problem Statement /objective of the study/research questions	Guiding conceptual or theoretical framework	Study design	Strength of study design	Sample and setting	Simulation development	Description of simulation implementation	Description of simulation feedback or debriefing	Study instrum[illegible] s
**G. M. Hughes et al (2013)[1]**	**3**	**2**	**3**	**2**	**2**	**2**	**3**	**3**	**3**	**2**	**2**
**W. Brodsky et al (2013)[2]**	**3**	**3**	**3**	**2**	**3**	**2**	**3**	**2**	**3**	**2**	**3**
**J. Navarro et al (2018)[3]**	**3**	**2**	**3**	**2**	**2**	**2**	**2**	**2**	**3**	**2**	**3**
**B. Dalton et al (2007)[4]**	**3**	**3**	**3**	**2**	**2**	**3**	**3**	**2**	**3**	**2**	**2**
**C. I. Karageorghis et al (2022)[5]**	**4**	**4**	**4**	**4**	**3**	**3**	**3**	**2**	**2**	**3**	**4**
**H. C. Beh et al (1999)[6]**	**3**	**2**	**2**	**2**	**2**	**2**	**2**	**2**	**2**	**1**	**2**
**J. Navarro et al (2019)[7]**	**2**	**1**	**1**	**1**	**2**	**1**	**1**	**2**	**2**	**2**	**2**
**A. B. Ünal et al (2013)[8]**	**2**	**2**	**2**	**2**	**2**	**3**	**3**	**2**	**3**	**3**	**2**
**D. B. Bellinger et al (2009)[9]**	**3**	**4**	**4**	**4**	**3**	**3**	**2**	**2**	**2**	**2**	**2**
**Z. N. Jimison (2014)[10]**	**3**	**3**	**3**	**4**	**3**	**4**	**2**	**2**	**2**	**2**	**2**
**A. Febriandirza et al (2017)[11]**	**3**	**2**	**3**	**3**	**3**	**3**	**2**	**2**	**2**	**2**	**2**
**G. G. Cassidy et al (2010)[12]**	**3**	**2**	**3**	**2**	**2**	**3**	**2**	**2**	**2**	**2**	**3**
**G. Cassidy et al (2009)[13]**	**3**	**3**	**3**	**2**	**3**	**2**	**2**	**2**	**2**	**2**	**2**
**W. Brodsky (2001)[14]**	**3**	**4**	**2**	**3**	**3**	**2**	**3**	**3**	**3**	**3**	**3**

**A. B. Ünal et al (2012)[15]**	**3**	**3**	**3**	**2**	**3**	**2**	**3**	**2**	**2**	**2**	**3**
**L. Miao et al (2021)[16]**	**3**	**4**	**3**	**3**	**3**	**3**	**3**	**2**	**2**	**1**	**4**

1. Hughes GM, Rudin-Brown CM, Young KL. A simulator study of the effects of singing on driving performance. *Accid Anal Prev.* 2013; 50: 787-92. doi:10.1016/j.aap.2012.07.001
2. Brodsky W, Slor Z. Background music as a risk factor for distraction among young-novice drivers. *Accid Anal Prev.* 2013; 59: 382-93. doi:10.1016/j.aap.2013.06.022
3. Navarro J, Osiurak F, Reynaud E. Does the Tempo of Music Impact Human Behavior Behind the Wheel? *Hum Factors.* 2018; 60(4): 556-74. doi:10.1177/0018720818760901
4. Dalton B, Behm D, Kibele A. Effects of sound types and volumes on simulated driving, vigilance tasks and heart rate. *Occupational Ergonomics.* 2007; 7: 153-68. doi:10.3233/OER-2007-7302
5. Karageorghis CI, Payre W, Howard LW, Kuan G, Mouchlianitis E, Reed N*, et al.* Influence of music on driver psychology and safety-relevant behaviours: a multi-study inductive content analysis. *TheoreTical issues in ergonomics science.* 2022; 23(6): 643-62. doi:https://doi.org/10.1080/1463922X.2021.2009933
6. Beh HC, Hirst R. Performance on driving-related tasks during music. *Ergonomics.* 1999; 42(8): 1087-98. doi:10.1080/001401399185153
7. Navarro J, Osiurak F, Gaujoux V, Ouimet MC, Reynaud E. Driving Under the Influence: How Music Listening Affects Driving Behaviors. *J Vis Exp.* 2019; (145). doi:10.3791/58342
8. Ünal AB, de Waard D, Epstude K, Steg L. Driving with music: Effects on arousal and performance. *Transportation Research Part F: Traffic Psychology and Behaviour.* 2013; 21: 52-65. doi:https://doi.org/10.1016/j.trf.2013.09.004
9. Bellinger DB, Budde BM, Machida M, Richardson GB, Berg WP. The effect of cellular telephone conversation and music listening on response time in braking. *Transportation Research Part F: Traffic Psychology and Behaviour.* 2009; 12(6): 441-51. doi:10.1016/j.trf.2009.08.007
10. Jimison ZN. The effect of music familiarity on Driving: A simulated study of the impact of music familiarity under different driving conditions. 2014.
11. Febriandirza A, Chaozhong W, Zhong M, Hu Z, Zhang H. The Effect of Natural Sounds and Music on Driving Performance and Physiological. *Engineering letters.* 2017 25(4).
12. Cassidy GG, Macdonald RA. The effects of music on time perception and performance of a driving game. *Scand J Psychol.* 2010; 51(6): 455-64. doi:10.1111/j.1467-9450.2010.00830.x
13. Cassidy G, Macdonald R. The effects of music choice on task performance: A study of the impact of self-selected and experimenter-selected music on driving game performance and experience. *Musicae Scientiae.* 2009; 13(2): 357-86. doi:10.1177/102986490901300207
14. Brodsky W. The effects of music tempo on simulated driving performance and vehicular control. *Transportation Research Part F: Traffic Psychology and Behaviour.* 2001; 4(4): 219-41. doi:10.1016/S1369-8478(01)00025-0
15. Ünal AB, Steg L, Epstude K. The influence of music on mental effort and driving performance. *Accid Anal Prev.* 2012; 48: 271-8. doi:10.1016/j.aap.2012.01.022
16. Miao L, Gu Y, He L, Wang H, Schwebel DC, Shen Y. The influence of music tempo on mental load and hazard perception of novice drivers. *Accid Anal Prev.* 2021; 157: 106168. doi:10.1016/j.aap.2021.106168
